# *Campylobacter jejuni* in Penguins, Antarctica

**DOI:** 10.3201/eid1505.081160

**Published:** 2009-05

**Authors:** Petra Griekspoor, Björn Olsen, Jonas Waldenström

**Affiliations:** Kalmar University, Kalmar, Sweden (P. Griekspoor, B. Olsen, J. Waldenström); Uppsala University, Uppsala, Sweden (B. Olsen)

**Keywords:** Zoonoses, bacteria, Campylobacter jejuni, penguins, Antarctica, enteric infections, letter

**To the Editor:** The wildlife of Antarctica is highly specialized. Although large animal species are limited primarily to penguins and seals, each species is often abundant. The high degree of isolation potentially protects Antarctic wildlife from diseases distributed in other areas of the world (*1*,*2*). Despite Antarctica’s isolation, however, human- or animal-related pathogens have been found there, or in the sub-Antarctic islands. For instance, serologic evidence of influenza virus A infections in penguins has been found (*3*), and both *Salmonella* spp. and *Mycobacterium tuberculosis* have been isolated from sub-Antarctic and Antarctic animals (*4*,*5*).

*Campylobacter jejuni* is a leading cause of bacterial gastroenteritis in humans worldwide; it is usually found in the intestinal tract of various farm and wild animals, particularly birds (*6*,*7*).We previously reported finding 3 *C. jejuni* subsp. *jejuni* isolates in macaroni penguins (*Eudyptes chrysolophus*; Figure) from Bird Island (54°00′S, 38°02′W), South Georgia (*1*). Phenotypic tests and 16S rRNA gene sequencing showed that the penguin isolates were identical to each other, and macrorestriction profiling of pulsed-field gel electrophoresis fragments showed that they were very similar to fragments isolated from poultry in Washington in 1984 (*1*). Because the isolates were retrieved from macaroni penguin chicks, we concluded that the animals had acquired the infection locally and that this was likely an instance of introduction of a pathogen to the Antarctic region.

**Figure:**
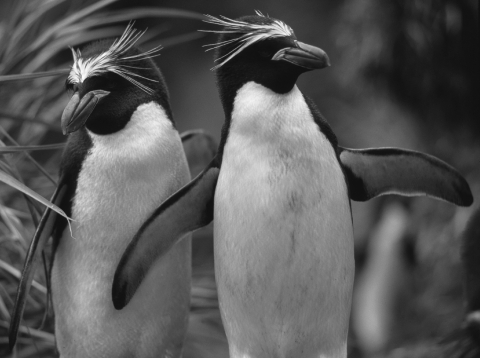
Macaroni penguins (*Eudyptes chrysolophus*). Photo by Jonas Bonnedahl.

However, restriction fragment pattern resemblance is not identical to genetic relatedness and, given the relevance of the question of origin, this resemblance led us to use a new method for genetic characterization. We reanalyzed the macaroni penguin isolates with multilocus sequence typing (MLST), a method that uses sequence data from 7 unlinked loci for genetic identification (*8*), complemented with *flaA* gene sequencing. A benefit of this method is the increasing availability of epidemiologic databases in which isolates can be compared (e.g., http://pubmlst.org/cam,pylobacter). The isolates were thawed and cultured on conventional blood agar (Columbia agar II containing 8% [vol/vol] whole horse blood) at 42°C in a microaerobic gas environment, with the CampyGen gas-generating system (CN0025A; Oxoid Ltd, Basingstoke, UK) and the BBL GasPak system (BD, Franklin Lakes, NJ, USA). Bacterial DNA was prepared by making a suspension of freshly grown bacterial cells in 200 µL of phosphate-buffered saline (Sigma, St. Louis, MO, USA). Genomic DNA was extracted by use of a Bio Robot M48 (QIAGEN, Hilden, Germany) with a MagAttract DNA mini M48 kit, according to the instructions of the manufacturer. The PCR amplification and nucleotide sequencing followed the original protocol in principle (*8*). The amplification products were purified and sequenced by using internal separated nested primer pairs.

The 3 isolates from macaroni penguins were all of the same genotype (sequence type [ST]–45) and thus have a common origin. The ST-45 sequence type is the central genotype of the ST-45 clonal complex, a complex often associated with human disease and asymptomatic infection in poultry (*9*,*10*). Indeed, nearly 42% of the ST-45 samples available in the MLST database have been isolated from humans (31% from poultry), and similar percentages have been observed for the ST-45 clonal complex as a whole. The ST-45 clonal complex is large (composed of 195 individual STs) and has been isolated to date from a variety of environmental sources and different geographic regions, with the exception of the Arctic. The isolates were identical at the *flaA* locus, all having the allele 21/peptide 2 designation (http://pubmlst.org/campylobacter/flaA). This particular peptide is found in 31 records in the database and is thus not unique to the penguin isolates.

Our MLST analysis confirms that the *C. jejuni* isolates from the penguins were of a genotype common among humans with disease and among our food animals. *C. jejuni* is not normally distributed among Antarctic animals (*1*,*2*), which indicates that this strain may have been imported through human activities. On Bird Island, such activities were carried out by scientists at the British Antarctic Survey base. At the time of the study, toilet wastes from the station were emptied into the surrounding waters, providing a possible transmission route for human-associated *C. jejuni* to reach wildlife, including penguins. Other possible sources of the *C. jejuni* infections include wastes from passing ships or seabirds that pick up the bacteria during offshore feeding excursions (for albatrosses, these can be ≈1,000 km). Once established in a penguin colony, a gastrointestinal pathogen may be transmitted rapidly among individual birds as they are breeding densely and producing a large amount of feces (guano) in the colony. *C. jejuni* infection in birds is normally not associated with overt disease, but other and possibly more devastating pathogens introduced to Antarctic animals could potentially cause outbreaks.
